# Enhanced plasticity of mature granule cells reduces survival of newborn neurons in the adult mouse hippocampus

**DOI:** 10.19185/matters.201610000014

**Published:** 2016-12-29

**Authors:** Felix B Kleine Borgmann, Johannes Gräff, Isabelle M Mansuy, Nicolas Toni, Sebastian Jessberger

**Affiliations:** Laboratory of Neural Plasticity, Brain Research Institute, University of Zurich, Luxembourg Centre for Systems Biomedicine, University of Luxembourg; Brain Research Institute, Laboratory of Neuroepigenetics, University of Zurich and Swiss Federal Institute of Technology Zurich, Department of Fundamental Neuroscience, University of Lausanne; Laboratory of Neuroepigenetics, Brain Research Institute, University of Zurich and Swiss Federal Institute of Technology Zurich; Departement des neurosciences fondamentales, Universite de Lausanne; Laboratory of Neural Plasticity, Brain Research Institute, University of Zurich

**Keywords:** Synaptic Integration, Synapses, Neurogenesis, Hippocampus, Synaptic Transmission

## Abstract

Dentate granule cells are born throughout life in the mammalian hippocampus. The integration of newborn neurons into the dentate circuit is activity-dependent, and structural data characterizing synapse formation suggested that the survival of adult-born granule cells is regulated by competition for synaptic partners. Here we tested this hypothesis by using a mouse model with genetically enhanced plasticity of mature granule cells through temporally controlled expression of a nuclear inhibitor of protein phosphatase _1_ (NIPP_1_*). Using thymidine analogues and retrovirus-mediated cell labeling, we show that synaptic integration and subsequent survival of newborn neurons is decreased in NIPP_1_*-expressing mice, suggesting that newborn neurons compete with preexisting granule cells for stable integration. The data presented here provides experimental evidence for a long-standing hypothesis and suggest cellular competition as a key mechanism regulating the integration and survival of newborn granule cells in the adult mammalian hippocampus.

## Introduction

Neural stem/progenitor cells (NSPCs) generate the vast majority of neurons in the brain during embryonic development. However, the neurogenic capacity of NSPCs does not end with birth as new neurons are born across the entire life-span in discrete areas of the mammalian brain [1]. One of these regions is the hippocampal dentate gyrus (DG) where NSPCs persist throughout life and continuously generate new granule cells, the principal neuronal subtype of the DG [2] [3]. Newborn neurons are critically involved in a number of hippocampus-dependent cognitive functions including behavioral pattern separation, forgetting, and mood regulation [4] [5] [6] [7] [8]. Moreover, failing or altered neurogenesis has been associated with several diseases such as major depression and epilepsy, suggesting that the addition of new neurons into the DG circuitry has translational relevance [9] [10] [11]. Similar to embryonic development, a surplus of neurons is initially generated in the adult brain. The survival and stable integration of new neurons appears to be regulated in a two-step process: whereas a substantial number of newborn neurons dies within the first days after their birth, there is a second phase of selection that occurs approximately 1-3 weeks after neuronal birth [12] [13] [14] [15]. Selection during this stage depends on N-methyl-D-aspartate (NMDA) receptor-mediated activity as conditional deletion of the GluN1 (NR1) subunit in newborn granule cells substantially decreases the number of surviving neurons [16]. During the phase of integration, newborn granule cells are highly excitable and remain so for approximately 6 weeks. This period of high cellular plasticity has been associated with unique functional properties of new neurons and may help new neurons to successfully integrate into the pre-existing circuit [17] [18] [19] [20] [21]. Indeed, it has been suggested that new neurons compete for synaptic partners allowing for stable integration and subsequent survival [22]. We here tested this hypothesis by examining whether the cell type-selective enhanced plasticity of mature granule cells affects integration and survival of newborn granule cells. Strikingly, we have found that enhancing the plasticity of the mature dentate granule cell circuit leads to decreased survival of newborn granule cells, providing experimental evidence that competition may be a critical component for the survival of newborn granule cells.

Neural stem/progenitor cells (NSPCs) generate the vast majority of neurons in the brain during embryonic development. However, the neurogenic capacity of NSPCs does not end with birth as new neurons are born across the entire life-span in discrete areas of the mammalian brain [1]. One of these regions is the hippocampal dentate gyrus (DG) where NSPCs persist throughout life and continuously generate new granule cells, the principal neuronal subtype of the DG [2] [3]. Newborn neurons are critically involved in a number of hippocampus-dependent cognitive functions including behavioral pattern separation, forgetting, and mood regulation [4] [5] [6] [7] [8]. Moreover, failing or altered neurogenesis has been associated with several diseases such as major depression and epilepsy, suggesting that the addition of new neurons into the DG circuitry has translational relevance [9] [10] [11]. Similar to embryonic development, a surplus of neurons is initially generated in the adult brain. The survival and stable integration of new neurons appears to be regulated in a two-step process: whereas a substantial number of newborn neurons dies within the first days after their birth, there is a second phase of selection that occurs approximately 1-3 weeks after neuronal birth [12] [13] [14] [15]. Selection during this stage depends on N-methyl-D-aspartate (NMDA) receptor-mediated activity as conditional deletion of the GluN1 (NR1) subunit in newborn granule cells substantially decreases the number of surviving neurons [16]. During the phase of integration, newborn granule cells are highly excitable and remain so for approximately 6 weeks. This period of high cellular plasticity has been associated with unique functional properties of new neurons and may help new neurons to successfully integrate into the pre-existing circuit [17] [18] [19] [20] [21]. Indeed, it has been suggested that new neurons compete for synaptic partners allowing for stable integration and subsequent survival [22]. We here tested this hypothesis by examining whether the cell type-selective enhanced plasticity of mature granule cells affects integration and survival of newborn granule cells. Strikingly, we have found that enhancing the plasticity of the mature dentate granule cell circuit leads to decreased survival of newborn granule cells, providing experimental evidence that competition may be a critical component for the survival of newborn granule cells.

## Objective

We investigate the survival and morphology of newborn granule cells in the adult dentate gyrus in a model of enhanced plasticity of mature neurons to test the hypothesis that a mechanism of competition governs integration into the neuronal circuits.

## Results & Discussion

### Expression of a nuclear inhibitor of PP_1_ (NIPP_1_*) in mature granule cells

**Figure 1:**
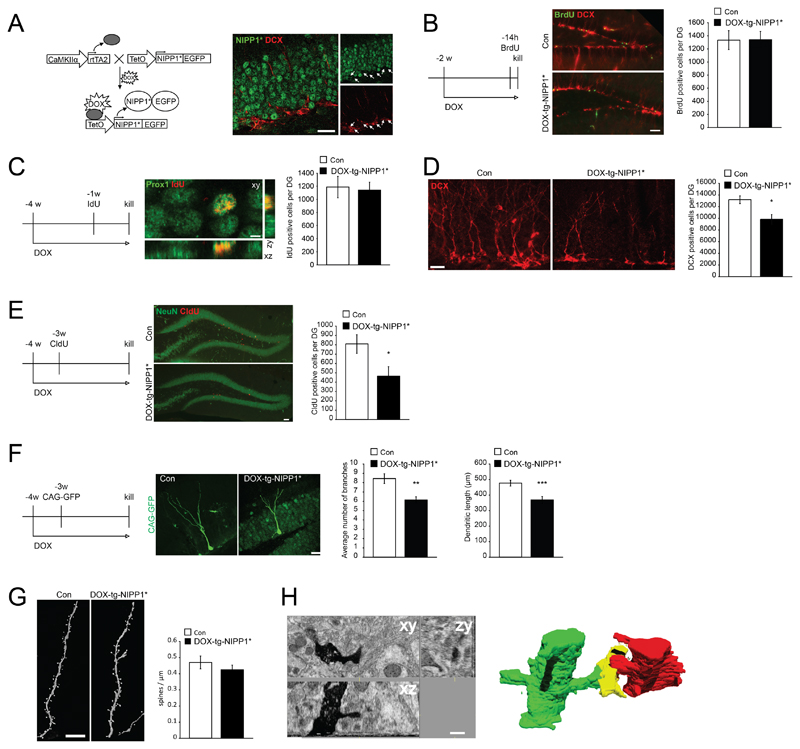
(A-C) CaMKII-driven NIPP_1_* expression in mature granule cells does not affect NSPC proliferation. **(A)** Genetic approach for conditional, DOX-regulated expression of NIPP_1_* in mature granule cells. Note that transgene-expressed nuclear GFP (green) is not expressed in newborn, DCX-expressing cells (red, arrows). Scale bar represents 20 μm. **(B)** Number of proliferating, BrdU-labeled cells (green) is not changed upon DOX treatment in control mice (upper panel) compared to DOX-tg-NIPP_1_* mice (lower panel). Graphs show quantification. Scale bar represents 50 μm. **(C)** NIPP_1_* expression in mature granule cells does not affect early survival and neuronal fate choice as measured using IdU injections 1 week before analysis (shown is an example of an IdU-labeled (red), Prox1-positive (green) cell) in control compared to DOX-tg-NIPP_1_* mice. Scale bar represents 5 μm. Graphs show quantification. **(D-E) Enhanced plasticity of the mature granule cell circuit impairs survival of newborn neurons. (D)** The number of newborn neurons expressing DCX (red) is reduced in DOX-tg-NIPP_1_* mice (right panel) compared to control mice (left panel). Graphs show quantification of DCX-labeled cells per DG. Scale bar represents 20 μm. **(E)** The number of newborn neurons, labeled with CldU (red) and expressing NeuN (green), is also reduced as measured using CldU injections 3 weeks before analyses in DOX-tg-NIPP_1_* mice (lower panel) compared to control mice (upper panel). Scale bar represents 50 μm. Graphs show quantification of CldU-labeled cells per DG. * *p* <0.05. (F-H) NIPP_1_* expression in mature granule cells impairs integration of new-born neurons. (F) Reduced dendritic complexity of newborn neurons 3 weeks after birth that were labeled by retrovirus-based GFP expression (green) in DOX-tg-NIPP_1_* mice (right panel) compared to control mice (left panel). Scale bar represents 20 μm. Graphs show quantification of dendritic length (left) and branching points (right). **(G)** Number of spines as measured per μm dendritic length is not altered in DOX-tg-NIPP_1_* mice (right panel) compared to the control mice (left panel). Scale bar represents 5 μm. Graphs show quantification. **(H)** Neurons born in the DOX-tg-NIPP_1_* mice are capable of forming MSBs as analyzed using FIB-SEM. Upper panels show the 3D view of 3 week old newborn neurons identified by viral labeling. Lower image shows 3D reconstruction of a MSB formed by a newborn neuron (green) and an unlabeled granule cell (red) that form a synapse onto an axon (yellow). Scale bar represents 500 nm. **p <0.01, ***p <0.001.

To test for evidence of activity dependent competition for survival between new neurons and pre-existing granule cells, we used a mouse model where the plasticity of mature granule cells is enhanced through transgenic expression of a constitutively active form of the nuclear inhibitor of protein phosphatase _1_ (NIPP_1_*) [23] [24] [25] [26]. NIPP_1_* expression in mature granule cells has been previously shown to enhance synaptic plasticity of granule cells in the adult DG, leading, for example, to an increased amplitude of long-term potentiation (LTP) [25] [26]. To selectively direct NIPP_1_* expression to mature granule cells, we used a cell type specific approach where NIPP_1_* is expressed under the control of the CaMKIIα promoter and the reverse tetracycline (Tet)-controlled trans-activator _2_ (CaMKIIα-driven rtTA_2_ x TetO-NIPP_1_*/EGFP; hereafter called tg-NIPP_1_*; Fig. 1A) [27] [25]. We found that the CaMKIIα promoter is not active in newborn granule cells expressing the microtubule-associated protein doublecortin (DCX) that is transiently expressed in newborn neurons but not in mature granule cells [28], and that transgene expression upon Doxycycline (DOX) treatment was highly selective in mature granule cells (NeuN^+^, DCX^-^) in the DG as measured by expression of EGFP fused to NIPP_1_* (Fig. 1A, and data not shown). Thus, transgenic NIPP_1_* expression within the DG is restricted to mature granule cells past the expression stage of DCX [28] making this model suitable for testing the effects of enhanced mature granule cell plasticity on NSPC proliferation, fate determination, and stable integration of newborn neurons.

### Tg-NIPP_1_* expression in mature granule cells does not affect NSPC proliferation

As previous reports suggested that NSPC proliferation is regulated by neuronal activity [29] [30] [31], we first analyzed if NSPC proliferation is affected upon tg-NIPP_1_* expression. We compared DOX-fed tg-NIPP_1_* mice with their single transgenic (lacking the TetO-NIPP_1_*/EGFP transgene) and non-DOX-fed tg-NIPP_1_* littermates as controls. Non-DOX-fed single as well as double transgenic mice and DOX-fed single and double transgenic mice were analyzed separately to exclude leakiness of the transgene expression and to test for a potential influence of DOX alone. We did not observe significant differences in the rate of proliferation and number of newborn neurons as measured using DCX expression and thymidine analogue labeling (BrdU, CldU, and IdU) between any of the control groups in any experiment (Suppl. Fig. 1A, B). Upon 2 weeks of DOX treatment, NSPC proliferation was analyzed using a single BrdU pulse (Fig. 1B) 14 h prior to killing the animals. Using this approach, we observed no significant difference between DOX-fed tg-NIPP_1_* and their respective controls (Con: 1332 ± 145; DOX-tg-NIPP_1_*: 1340 ± 125 BrdU^+^ cells per DG, n.s). We next analyzed if tg-NIPP_1_* expression affects early neuronal cell death or fate specification of newborn cells. Using the thymidine analogue IdU, we labeled cells 1 week prior to analyses and found no difference between tg-NIPP_1_* mice and respective controls (Fig. 1C) (Con: 1184 ± 163; DOX-tg-NIPP_1_*: 1138 ± 122 IdU^+^ cells per DG, n.s.). In addition, we found that virtually all IdU-labeled cells expressed the neuronal markers DCX and Prox1 (Fig. 1C, and data not shown), suggesting that fate determination and differentiation of newborn neurons is not affected in DOX-tg-NIPP_1_* mice.

### Enhanced plasticity of the mature granule cell circuit impairs survival of newborn neurons

We then tested if the total number of immature neurons in tg-NIPP_1_* mice is affected upon DOX treatment. DCX starts to be expressed in late dividing NSPCs and expression lasts for approximately 3 weeks after neuronal birth [32] [28]. Strikingly, the number of DCX-expressing cells was substantially reduced in DOX-tg-NIPP_1_* mice (Con: 13182 ± 662.2; DOX-tg-NIPP_1_* 9810 ± 828.8 DCX^+^ cells per DG, *p* <0.05), indicating a loss of immature neurons at later stages when activity dependent survival occurs (Fig. 1D) [16]. We used a complementary approach to confirm the loss of new neurons based on DCX analyses and injected the thymidine analogue CldU in DOX-tg-NIPP_1_* mice and respective controls. Animals were killed 3 weeks later and the number of CldU^+^ cells analyzed. Corroborating the DCX results, we found a significant drop in CldU-labeled cells in DOX-tg-NIPP_1_* mice compared to controls (Con: 808 ± 100 control; DOX-tg-NIPP_1_*: 465 ± 101 CldU^+^ cells per DG, *p* <0.05) (Fig. 1E). Interestingly, these results suggest that neuronal loss occurs around the time when newborn neurons start to form dendritic spines and excitatory synapses [33] [22] suggesting that synaptic competition, impaired by enhanced plasticity of the mature granule circuit through NIPP_1_* expression, may be critical for stable integration into the DG network.

### Reduced dendritic complexity in newborn neurons in tg-NIPP_1_* mice

We next analyzed if the length and branching of newborn neurons are affected in DOX-tg-NIPP_1_* mice, using these measures as a proxy for neuronal integration [34]. To analyze the morphology of newborn neurons, we injected retroviruses expressing GFP under chicken beta-actin promoter stereotactically into the DG of control and tg-NIPP_1_* mice and killed the animals 3 weeks later [33]. Using this approach, we found that dendritic length was significantly reduced in DOX-tg-NIPP_1_* mice compared to controls (Fig. 1F) (Con: 476.8 μm ± 18.5; DOX-tg-NIPP_1_*: 368.1 μm ± 22.8 average dendritic length, *p* <0.001). Further, dendrites extending from neurons born in DOX-tg-NIPP_1_* mice had substantially fewer branches compared to controls (Fig. 1F) (Con: 8.43 ± 0.52; DOX-tg-NIPP_1_*: 6.14 ± 0.35, branch points per neuron, *p* <0.01). In contrast, we found that axonal growth into area CA_3_, which is reached by axons extending from newborn neurons before first spines are formed [33] was not altered in DOX-tg-NIPP_1_* mice (Suppl. Fig. 2) (Con: 1122.74 ± 91.73 μm; DOX-tg-NIPP_1_* 1225.73 ± 42.36 μm, n.s.). After finding that dendrites extending from newborn neurons in DOX-tg-NIPP_1_* were shorter and less complex, we next analyzed the number of dendritic spines, the main place for excitatory synapses of excitatory neurons, in DOX-tg-NIPP_1_* and control mice (Fig. 1G). On the dendritic segments analyzed, the number of spines per μm dendrite was similar between groups (Con: 0.469 ± 0.04; DOX-tg-NIPP_1_*: 0.423 ± 0.02 spines/μm, n.s.) (Fig. 1G [22]). Furthermore, the subtype of spines did not differ between DOX-tg-NIPP_1_* and control mice (Con: 0.761 ± 0.07; DOX-tg-NIPP_1_*: 0.898 ± 0.07 mushroom spines/μm; Con: 0.136 ± 0.01; DOX-tg-NIPP_1_*: 0.105 ± 0.09 stubby spines/μm; Con: 0.256 ± 0.03; DOX-tg-NIPP_1_*: 0.227 ± 0.02 thin spines/μm, n.s.). However, given that dendrites are shorter in DOX-tg-NIPP_1_* mice, we reasoned that despite similar spine density the total number of excitatory inputs is reduced per newborn neuron in DOX-tg-NIPP_1_* mice compared to controls. To estimate the number of spines per cell, we multiplied spines per μm with the calculated dendritic length. It has to be considered, however, that spines are not uniformly distributed on the dendrites; so any calculations can only be a rough estimation (e.g., within the granule cell layer (GCL), only few spines are formed). Given that dendrites are substantially longer in controls than in DOX-tg-NIPP_1_* mice, we estimated a reduction of synaptic input of approximately 50% of newborn neurons in DOX-tg-NIPP_1_* compared to controls (195.7 in control, 130.6 in DOX-tg-NIPP_1_* calculated spines per cell). Next, we analyzed synapse formation of new neurons in DOX-tg-NIPP_1_* on the ultrastructural level [22]. Again, we used retroviruses expressing GFP to label newborn neurons in DOX-tg-NIPP_1_* mice and controls. Synapse formation was analyzed 3 weeks after stereotactic injection of retroviruses and after immunhistochemical conversion of the GFP signal into osmiophilic DAB precipitate. We then used focused ion beam scanning (FIBS)- electron microscopy (EM) to reconstruct spines and their environment in 3-dimensions (*n* = 1 mouse per genotype). In total, we analyzed 73.2 μm of 2 dendrites containing 36 synapses in DOX-tg-NIPP_1_* (*n* = 1). Using this approach, we found that spines of newborn neurons in DOX-tg-NIPP_1_* mice formed synapses with visible postsynaptic densities, indicating functional connectivity, and engaged in multiple synapse boutons (MSBs), suggesting that synapse formation is not fundamentally altered in DOX-tg-NIPP_1_* mice (Fig. 1H) [22].

We used a genetic approach to test if the survival of new neurons in the adult DG is influenced by the plasticity of the mature granule cell circuit. Strikingly, we found that dendritic integration and subsequent survival of newborn granule is impaired with enhanced plasticity of the mature granule cell circuit, suggesting that competition for synaptic integration is critical in regulating the survival of new neurons. New neurons need to receive synaptic input for their survival, and structural data suggested that filopodia extending from dendrites of newborn neurons initially grow towards existing synapses between mature granule cells and presynaptic axons in the perforant path originating from neurons in the entorhinal cortex, leading to the formation of MSBs [16] [22]. With time, MSBs are then presumably exchanged by single synapse boutons (SSBs) suggesting that new neurons that successfully compete for synaptic partners stably integrate [22]. The survival of newborn neurons can be enhanced, for example, by environmental enrichment that appears to enhance excitability in the DG circuit [35] [36]. Similarly, the reduced survival of new neurons lacking the GluN1 subunit of the NMDA receptor can be partially rescued by global blockade of NMDA-dependent activity [16]. However, characterizing a potential competition by manipulating the balance of excitability selectively between new and mature granule cells had not been tested experimentally. We achieved this by cell type-specific expression of NIPP_1_* in mature granule cells and found that enhancing plasticity of the mature granule cell circuit [24] [25] impairs the survival of newborn granule cells. The growth of dendrites extending from newborn granule cells was substantially impaired in NIPP_1_*-expressing mice, whereas synapse formation appeared to be unaltered as analyzed using conventional light microscopy and electron microscopy. This led to a strongly reduced number of dendritic spines of newborn neurons, suggesting that their overall excitatory synaptic input is decreased. Since the transgene is expressed in all CaMKII-expressing cells, including those of the entorhinal cortex, we cannot exclude an effect of more globally changed activity of neuronal circuits. Without longitudinal imaging of synapse formation, which is currently technically not feasible at the required resolution, it cannot be proven that new neurons fail to survive due to impaired synaptic competition. However, our data strongly supports the hypothesis that new neurons need to compete for synaptic partners to ensure proper integration and survival [22]. Interestingly, it has been shown that during the first 3−6 weeks after their birth, new granule cells are highly excitable and show a higher degree of plasticity compared to mature granule cells [17] [18] [19] [21]. This unique feature has been attributed to their special functional properties with emerging evidence supporting the idea that adult neurogenesis in the DG is not a process for mere cell replacement but that new neurons exert their function at least partially due to these special properties [37] [38] [39]. However, it is also reasonable to speculate that the phase of heightened excitability may also be important to ensure the integration of new neurons, giving them a competitive advantage to form synapses with axons arising from the entorhinal cortex. Our data supports this idea by showing that the survival of new neurons is impaired with increased plasticity of the mature granule cell circuit, whereas NSPC proliferation and initial steps of fate determination and specification are unaltered. Thus, we here experimentally support a long-standing hypothesis, indicating that synaptic competition represents a key mechanism regulating the integration and survival of newborn granule cells in the adult mammalian hippocampus.

## Limitations

We used a single mouse model of enhanced plasticity with wildtype littermate controls. While, we interpret our data as a strong suggestion of a competitive mechanism at work, additional studies using different models and approaches are needed to prove that the effects observed are not specific to the model. In our model, the transgene is expressed under the control of the CaMKIIα promoter, which is active in all forebrain neurons. It is possible that, next to the effect of the immediate environment, other changes inherent to the transgene also affect neuronal integration and morphology. One immediate effect on newborn granule cells may be altered input by medial entorhinal cortex cells, the primary input of the hippocampus.

## Supplementary Material

Suppl. Fig.
