# Supplementary material for: Enhanced plasticity of mature granule cells reduces survival of newborn neurons in the adult mouse hippocampus
Source: Matters Sel. Author manuscript; Available in PMC 2022 Sep 26. (PMC7613637; doi:10.19185/matters.201610000014)
Supplement: Suppl. Fig. [file EMS146324-supplement-Suppl__Fig_.pdf]

Supplemental Figures

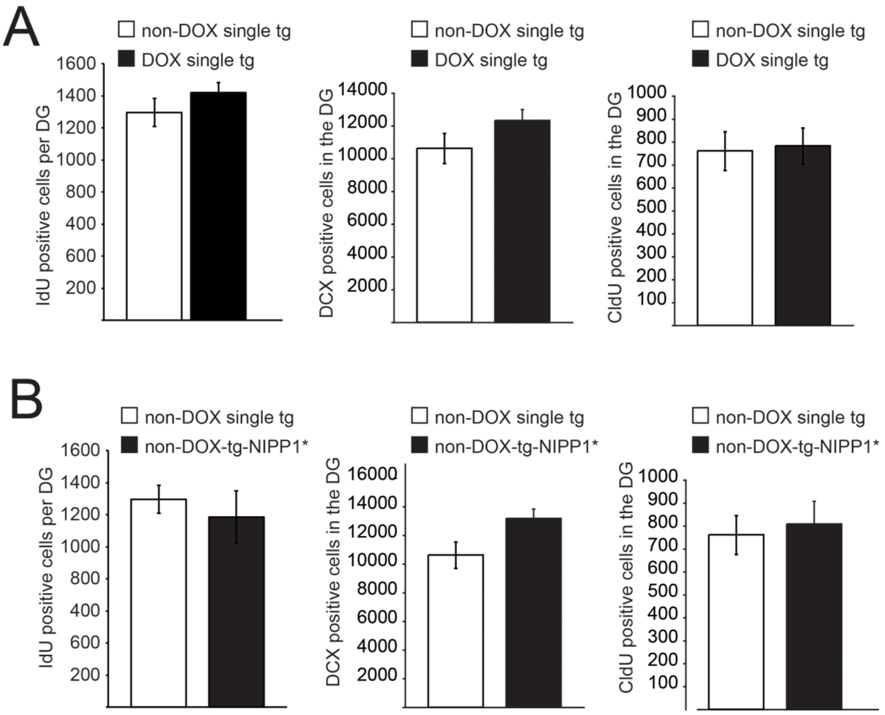

Supplemental Figure 1. Effects of DOX treatment and different genotypes on hippocampal neurogenesis.

(**A**) Effects on IdU-labeled, DCX-positive, and CldU-labeled cells in single transgenic mice (CaMKIIα-rtTA2 or TetO-NIPP1*/EGFP) mice non-treated (non-DOX) or treated with DOX. (**B**) Effects of genotype on IdU-labeled, DCX-positive, and CldU-labeled cells comparing single transgenic mice with tg-NIPP1* mice without DOX treatment (non-DOX).

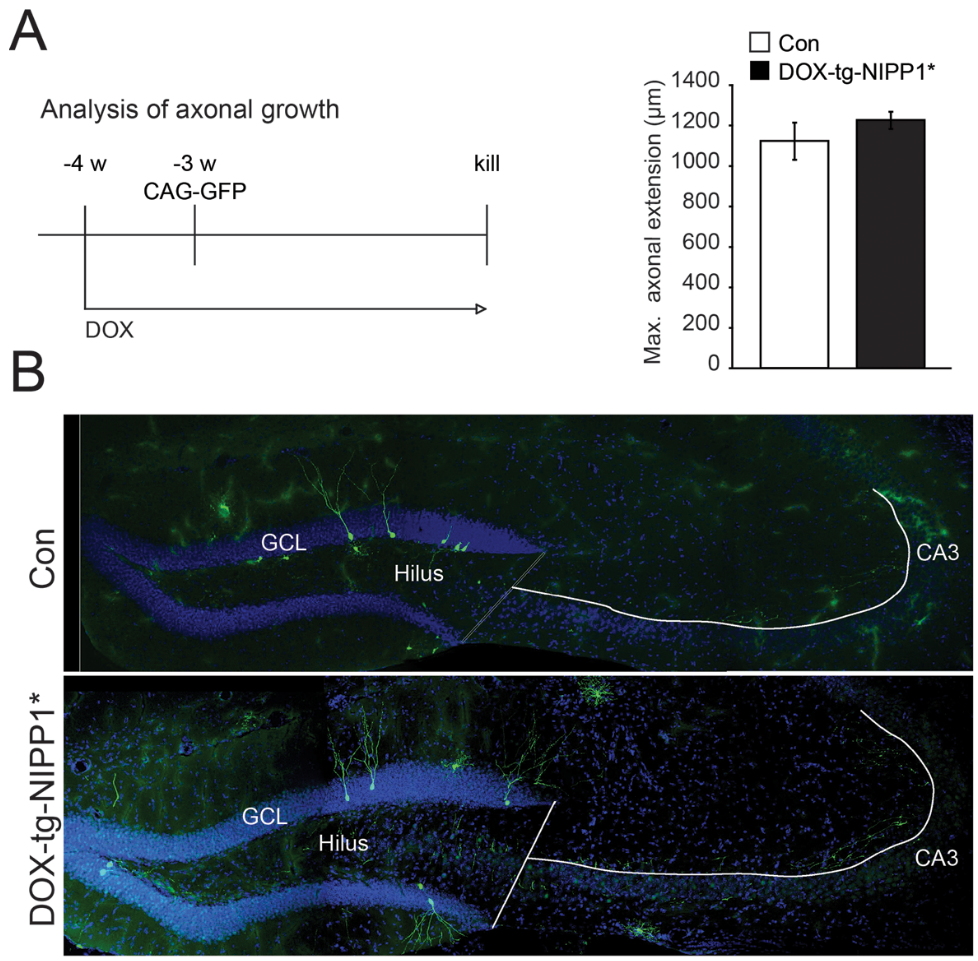

Supplemental Figure 2. Growth of axons extending from newborn neurons is not affected in DOX-tg-NIPP1* mice.

(**A**) Experimental setup to test for effects of axonal growth in DOX-tg-NIPP1* mice compared to controls. Graph shows the maximal extension of axons extending from 3 weeks old, GFP labeled neurons. (**B**) Representative images of 3 weeks old, GFP labeled neurons (green). Nuclei were counterstained with DAPI (blue).

GCL, granule cell layer; CA 3, cornu ammonis area 3.
